# Antimicrobial Activity of *Micrurus* Venoms and Bioactive Films Functionalized with Purified L-Amino Acid Oxidase

**DOI:** 10.3390/toxins18060240

**Published:** 2026-05-22

**Authors:** Vitelbina Núñez Rangel, Paola Rey-Suárez, Daniel Buitrago-Chinchilla, Laura Reyes-Méndez, Leidy Gómez-Sampedro, Alejandro Carmona-Jiménez, Mateo Rivillas-Ochoa, Adriana Muñoz-Bravo

**Affiliations:** 1 Grupo de Investigación en Toxinología, Alternativas Terapéuticas y Alimentarias (TOXATA), Universidad de Antioquia, Medellín 050010, Colombia; 2 Escuela de Microbiología, Universidad de Antioquia, Medellín 050010, Colombia; 3 Facultad de Ciencias Exactas y Aplicadas, Institución Universitaria ITM, Medellín 050034, Colombia; 4 Laboratorio de Investigación y Desarrollo Agroalimentario (LIDA), Escuela de Ciencias Básicas Tecnología e Ingeniería, Universidad Nacional Abierta y a Distancia, Bogotá 111511, Colombia; dbuitragoch@unadvirtual.edu.co (D.B.-C.); laura.reyes@unad.edu.co (L.R.-M.); leidyj.gomez@unad.edu.co (L.G.-S.); 5 Grupo de Investigación Biociencias, Institución Universitaria Colegio Mayor de Antioquia, Medellín 050034, Colombia; lacarmona@est.colmayor.edu.co (A.C.-J.); mrivillaso@est.colmayor.edu.co (M.R.-O.); adriana.bravo@colmayor.edu.co (A.M.-B.)

**Keywords:** *Micrurus*, antimicrobial, *Xanthomonas*, *Fusarium*, *M. dumerilii*, *M. mipartitus*, *M. ancoralis*, biofilms

## Abstract

Phytopathogenic bacteria and fungi significantly reduce fruit and vegetable yields, resulting in substantial economic losses. Conventional management relies on synthetic agrochemicals; however, their intensive use poses risks to human health, environmental integrity, and biodiversity. Snake venoms have evolved under selective pressure, developing specialized components with potent antimicrobial properties as part of a defense mechanism against prey-borne microorganisms. This study evaluated the inhibitory potential of *Micrurus* venoms against pathogens of agricultural interest and developed bioactive gelatin-based films incorporated with purified L-amino acid oxidases (LAAOs) as a novel biocontrol strategy. Venoms from *M. ancoralis*, *M. mipartitus*, and *M. dumerilii* exhibited significant growth inhibition against *Xanthomonas* and *Fusarium* strains. The primary active component was identified as LAAO through biological activity and mass spectrometry. Biofilms were formulated by incorporating *M. ancoralis* venom and its purified LAAO into a gelatin matrix. Physicochemical and microbiological characterization, alongside in situ assays on strawberries, demonstrated that the functionalized biofilms retained potent antimicrobial activity. Furthermore, LAAO incorporation did not significantly alter the physicochemical properties of the fruit but effectively extended shelf life by reducing weight loss and maintaining sensory appearance. These findings highlight the biotechnological potential of elapid venom components in the development of alternatives for phytopathogen control and active food packaging.

## 1. Introduction

The most important pathogens that cause significant damage to fruit and vegetable crops are bacteria and fungi, generating substantial annual economic losses [1,2,3]. Conventional management of these phytopathogens relies heavily on the application of agrochemicals with biocidal properties, including antibacterial, antifungal, and antiviral agents. Nevertheless, strains resistant to these have already been described, contributing to the selection of organisms that lead to diseases with a higher incidence than before [3].

Furthermore, the indiscriminate use of these substances constitutes a significant risk to human health, exacerbating environmental contamination and biodiversity loss [4,5]. Consequently, new molecules or strategies are needed that can be used to control these plant pathogens, thus contributing to reducing the economic losses that these microorganisms are generating in agriculture [5]. The crops are frequently affected by bacteria of the genus *Xanthomonas*, causing the disease called black rot or common blight [6,7], or by the fungus *Fusarium* spp., which causes vascular wilt [8,9]. Among these, *Xanthomonas axonopodis* and *X. perforans* are two of the major causal agents of bacterial blight in cassava and bacterial spots in tomato and pepper, respectively, leading to significant yield losses in economically important crops, particularly in South America. Furthermore, controlling soil-borne phytopathogens is very difficult, and plant diseases caused by microorganisms are among the most important factors that generate losses and affect crop yields. These microorganisms alter plants’ physiological functions, impairing their normal functioning, generally reducing yields, and, in extreme cases, leading to plant death [10].

It has been proposed that snake venom components evolved under selective pressure as a defense mechanism against microorganisms present in their prey [11]. There is a diversity of protein isoforms in venoms, indicating adaptations to prey and the environment [11]. Since Ondetti et al.’s discovery in 1971 of the peptides that gave rise to captopril from the venom of the *Bothrops jararaca* snake [12], interest has been sparked in animal venoms as rich sources of bioactive compounds and in the potential use of these compounds [13]. Different components had been studied for a wide range of pharmacological properties, including antimicrobial activity. Among the proteins described include lectins, metalloproteinases, L-amino acid oxidases, serine proteases, cysteine-rich proteins, phospholipase A_2_, and some small peptides [14,15,16], and the isolation and characterization of a novel peptide (PepBj) from *Bothropos jararaca* snake venom was reported, which was active against different fungi. In the same way, crotamine, a peptide with 42 amino acid residues isolated from rattlesnake venom, showed antibacterial activity against *E. coli* strains, and an analysis of the mechanism of action demonstrated that crotamine kills the bacteria by first penetrating the membrane [17]. A small protein of 50 amino acid residues was isolated from *Oxyuranus microlepidotus*, named omwaprin, and showed antimicrobial activity against Gram-positive bacteria, such as Bacillus magaterium and *Streptococcus warneri* [18]. Peptides synthesized from omwaprin demonstrated a broad spectrum and increased antimicrobial and antibiofilm activities [19]. Different snake venoms’ LAAO demonstrated the inhibition of different bacteria [20].

However, these venoms or isolated compounds can be sensitive to environmental conditions that can reduce their effectiveness [21]. Biopolymer-based films offer an innovative solution by serving three key functions: they act as physical barriers, enable controlled release of active compounds, and protect these molecules from degradation [22,23]. Gelatin-based films have demonstrated film-forming properties, biocompatibility, and the ability to incorporate antimicrobial peptides and other bioactive compounds into their polymeric matrix. The release properties of gelatin films can be modulated by modifying their composition, for instance, adding glycerol as a plasticizer affects not only mechanical strength and water permeability but also the rate of antimicrobial diffusion from the film [24]. When applied as edible coatings on fruits and vegetables, these films gradually release antimicrobial agents at the surface, effectively controlling phytopathogenic bacteria and fungi throughout storage [25].

Given the scientific evidence on the antimicrobial activity of compounds present in snake venom, this work aims to determine the antimicrobial effectiveness of venom of the *Micrurus* genus and their compounds against phytopathogenic fungal and bacterial strains that damage vegetable crops. Furthermore, the study evaluates the development and characterization of gelatin-based biofilms as functional delivery scaffolds for these bioactive compounds, providing a sustainable biotechnological approach for agricultural pathogen management.

## 2. Results

### 2.1. Antimicrobial Activity of Whole Venom and Fraction

Venoms from *M. ancoralis*, *M. dumerilii*, and *M. mipartitus* inhibited the growth of *Xanthomonas* strains in both agar diffusion and liquid broth assays, confirming their antimicrobial potential. This inhibitory effect was dose dependent across all tested species. The highest activity was observed for *M. ancoralis* against *X. axonopodis* and *X. perforans*, with MIC values of 20.8 ± 7.2 and 18 ± 13 µg/mL, respectively (Table 1).

**Figure 1:**
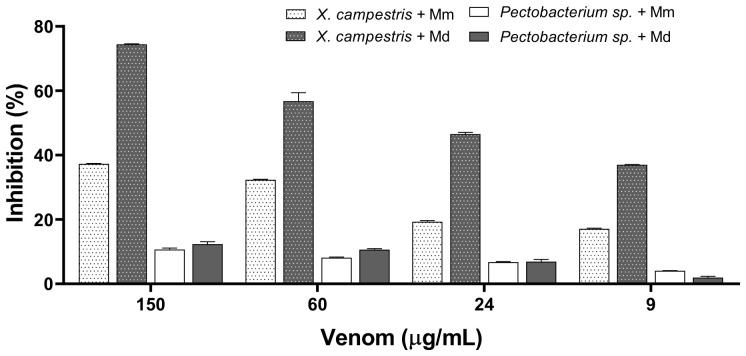
Antibacterial activity of *Micrurus mipartitus* (Mm) and *Micrurus dumerilii* (Md) crude venoms against phytopathogenic bacteria. Growth inhibition of *Xanthomonas campestris* (dotted bars) and *Pectobacterium* sp. (solid bars) was evaluated using different venom concentrations. A bacterial suspension without venom was used as the negative control to determine baseline growth. Each data point represents the mean ± SD of three independent experiments performed in triplicate (*n* = 3).

Furthermore, the venoms were also evaluated against *X. campestris* and *Pectobacterium* sp., the inhibitory activity was lower, and no total inhibition of microorganism growth was achieved at the evaluated doses (Figure 1).

**Figure 2:**
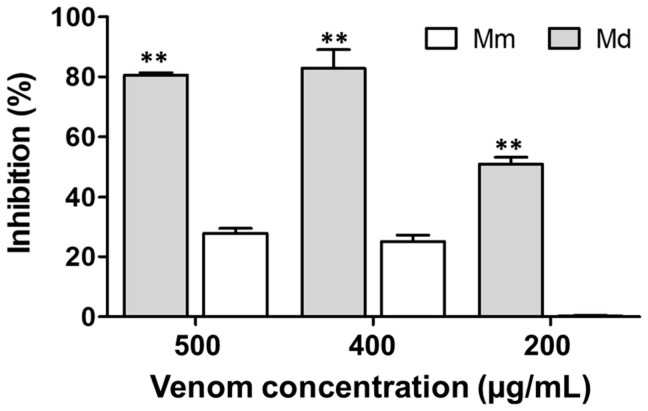
Antifungal activity of *Micrurus* venom against *Fusarium* sp. Growth inhibition assays was performed using *M. dumerilii* and *M. mipartitus* venoms. Data on the graph represents the mean ± SD of the triplicate (*n* = 3), showing statistical significance of percentage of inhibition each venom ** (*p* < 0.01).

In another assay, the *M. dumerilii* and *M. mipartitus* venoms were evaluated against the fungus *Fusarium* sp. The whole venoms evaluated showed inhibitory activity against *Fusarium* sp. at concentrations higher than those required to observe an antibacterial effect. Nevertheless, the venom of *M. dumerilii* showed greater inhibitory activity than *M. mipartitus* (Figure 2).

### 2.2. Isolation and Characterization of the Bioactive Fraction

**Figure 3:**
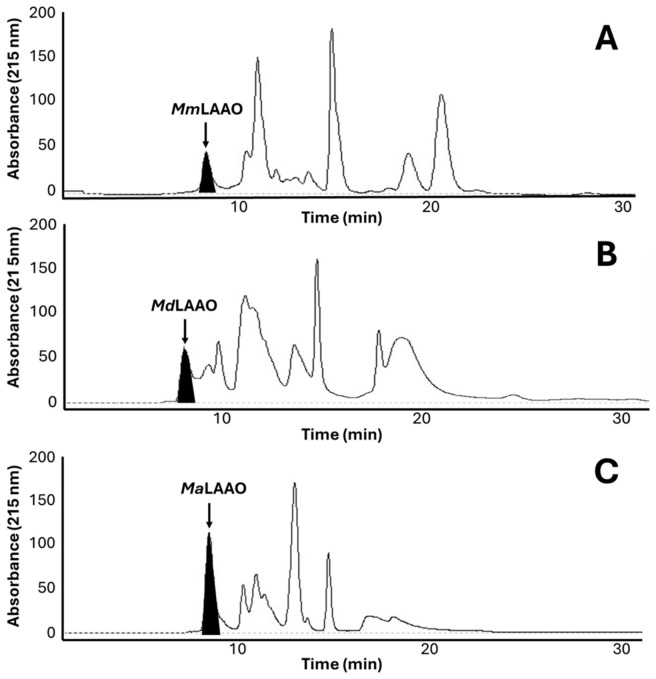
Size-exclusion chromatography (SEC) profile and isolation of bioactive fractions from *Micrurus* venoms: (**A**) *M. mipartitus* (*Mm*), (**B**) *M. dumerilii* (*Md*), and (**C**) *M. ancoralis* (*Ma*). Crude venom (2 mg) was fractionated using a Bio Sec-5 column on a BioRad DuoFlow™ chromatography system. The eluate was monitored by absorbance at 215 nm. Shaded peaks (black) indicate bioactive fraction identified as L-amino acid oxidase (LAAO). Identification was confirmed through both enzymatic activity assays and LC-MS/MS analysis.

**Figure 4:**
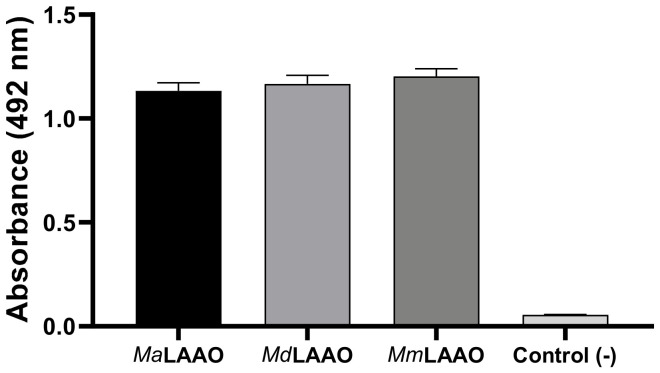
Enzymatic activity of isolated LAAOs from *Micrurus* venoms. The oxidative deamination of L-leucine (L-Leu) was measured to confirm the functional integrity of the enzymes post-purification. A reaction mixture containing the substrate and buffer without the enzyme was used as a negative control. Each data point represents the mean ± SD of three independent experiments performed in triplicate (*n* = 3).

Each venom was fractionated using size-exclusion chromatography (Figure 3), and the resulting fractions were collected and screened for antimicrobial activity against *Xanthomonas* species at concentrations equivalent to the previously determined MICs of the *Micrurus* venoms. Among all evaluated fractions, only the first fraction (F1) from each venom exhibited a growth inhibition halo against *X. perforans* and *X. axonopodis* in agar diffusion assays. This fraction of each venom was subsequently evaluated by the broth microdilution technique using different concentrations to determine the MIC for each one against these bacteria (Table 2). Functional assays confirmed that this bioactive fraction showed L-amino acid oxidase (LAAO) activity (Figure 4). Furthermore, de novo sequencing and subsequent multiple sequence alignment identified this protein as LAAO (Supplementary File S1).

### 2.3. Preparation of Films and Characterization

Films were prepared by incorporating either *M. ancoralis* crude venom or its purified LAAO (*Ma*LAAO). In both cases, the antimicrobial activity was successfully retained after the film-forming process, as well as their MIC values of 12.5 ± 4.8 µg/mL against both *X. axonopodis* and *X. perforans*.

Film properties such as thickness, moisture content, solubility in water, and aspects in UV/visible light or infrared were determined. The incorporation of the venom (Ma) or the fraction (*Ma*LAAO) does not significantly affect thickness, MC, and WS values (Table 3). SW shows no significant differences among the three samples (Gel, Gel + Ma, and Gel + *Ma*LAAO). A decrease in the MC of the films is evident with the incorporation of Ma and *Ma*LAAO, going from 11.7 ± 0.9% for Gel to 8.3 ± 0.4% for Gel + *Ma*LAAO. The film thickness increased slightly with the incorporation of *Ma*LAAO, from 0.046 ± 0.002 mm to 0.051 ± 0.004 mm.

In general, all films presented a light coloration (L* > 90), indicating clear and homogeneous surfaces, with no significant differences between formulations (*p* > 0.05) (Table 4). The a* parameter (red-green chroma) showed negative values close to zero, reflecting a slight tendency towards green. Meanwhile, the b* parameter showed positive values between 4.92 ± 0.2 for Gel and 5.68 ± 0.51 for Gel + *Ma*LAAO, indicating a slight yellow tint characteristic of protein matrices [26,27]. The opacity of the films was directly influenced by the addition of *Ma*LAAO, with gelatin films exhibiting values of 1.30 ± 0.03 and gelatin films with *Ma*LAAO increasing their opacity to 7.30 ± 0.8.

**Figure 5:**
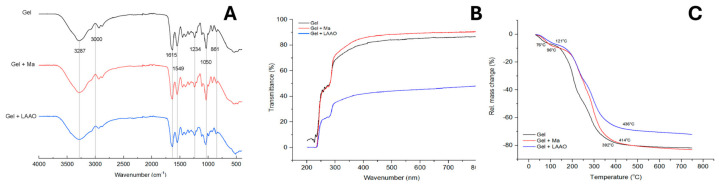
Properties of films. (**A**) UV/visible light barrier. (**B**) Fourier transform infrared (FTIR). (**C**) TGA curves (thermogravimetric analysis). Gel: film alone, Gel + Ma: film incorporated with *M. ancoralis* venom, and Gel + *Ma*LAAO: film incorporated with *M. ancoralis* LAAO.

This film presented a good UV barrier (T < 20%) to 300 nm (Figure 5A). Additionally, in the UV radiation range of 200 to 280 nm, all films exhibited excellent UV barrier properties, likely due to the presence of aromatic amino acids such as tyrosine and tryptophan in proteins, which absorb UV light around 280 nm [28,29].

The infrared spectra results (Figure 5B) of the three formulations analyzed show the main characteristic signals of gelatin-based biopolymeric materials, such as a band at 3287 cm^−1^ (N–H stretching of Amide A), associated with hydrogen bonds, as well as a signal at 3000 cm^−1^ corresponding to the asymmetric C–H stretching of methylene groups [27,30]. The intense bands at 1615.1 and 1549 cm^−1^ are assigned to Amide I (C=O) and Amide II (N–H and C–N), characteristic of proteins such as gelatin and venom peptides [30,31]. Signals were also identified at 1234 cm^−1^ (Amide III), 1050 cm^−1^ (C–O stretching, associated with residual carbohydrates), and 861 cm^−1^ (out-of-plane bending of aromatic C–H bonds).

Thermogravimetric analysis of the three films (Figure 5C) showed three stages of mass loss. The first stage occurred between 76 and 121 °C. The second stage was observed between 140 and 280 °C, with the third stage between 392 and 436 °C. The overall mass loss was similar for the Gel and Gel + Ma film, whereas the Gel + *Ma*LAAO formulation exhibited a comparatively lower total mass loss.

**Figure 6:**
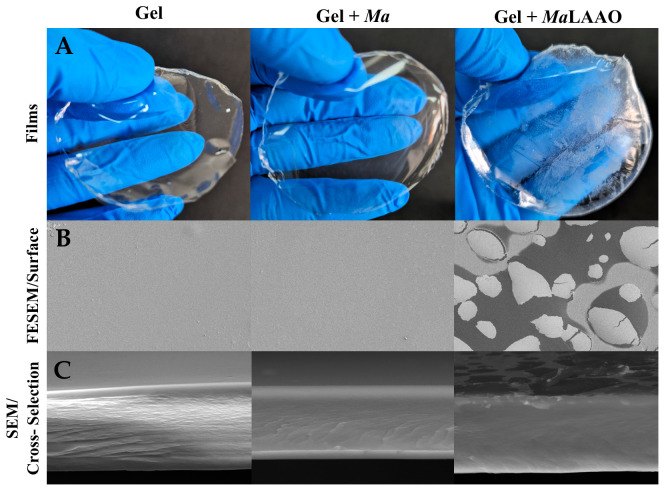
Macrostructure and microstructure of gelatin films. (**A**) Digital photographs, (**B**) FESEM images of the film surface (X 5000, scale 5 μm, 2 kV), and (**C**) SEM images of the cross-section of the films (X3000, scale 5 μm, 20 kV): Gel, Gel + Ma, and Gel + *Ma*LAAO.

Analysis of digital photographs and scanning electron microscopy images of the three film formulations (Gel, Gel + Ma, and Gel + *Ma*LAAO) showed significant differences in the surface and cross-sectional morphologies of Gelatin and Gelatin + Ma films versus Gelatin + *Ma*LAAO (Figure 6).

### 2.4. In Situ Films Tests

**Figure 7:**
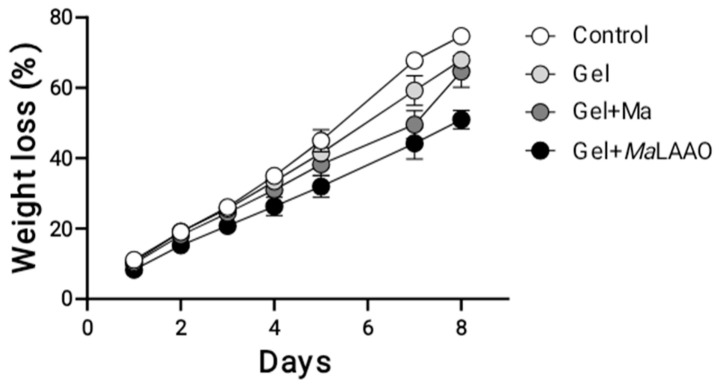
Relative weight loss values of strawberries with or without film. Control strawberries without film. After coating, strawberries were observed during storage for 8 days at ±20 °C. Each data point represents the mean ± SD of three independent experiments (*n* = 3).

Biodegradable films, with or without the incorporation of *M. ancoralis* crude venom or its purified fraction (*Ma*LAAO), were applied as coatings on strawberries to evaluate their effect on shelf life during storage at 20 °C and 55% relative humidity for 8 days. Figure 7 shows the relative weight loss (%) throughout the evaluation period. Uncoated strawberries (control) exhibited the greatest weight loss, reaching 74.7% on day 8, whereas all coated treatments significantly reduced mass loss, with the lowest value observed for the Gelatin + *Ma*LAAO treatment (50.9%), representing a reduction of more than 20% compared with the control.

**Figure 8:**
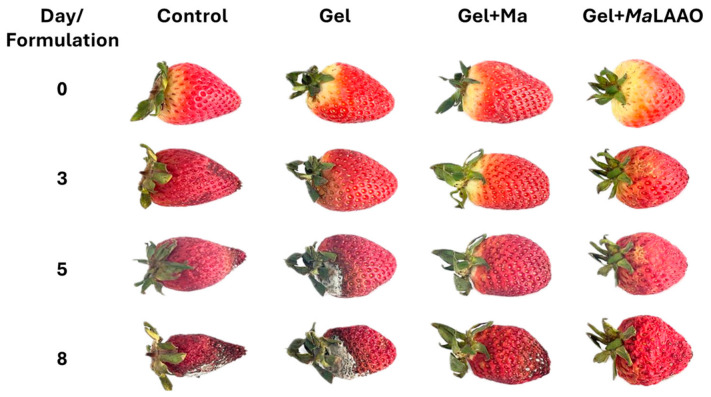
In situ films tests. Strawberries with or without film were evaluated in terms of visual appearance. Control strawberries without Gel, Gel alone, Gel + Ma, or Gel + *Ma*LAAO. After coating, strawberries were observed during storage for 8 days at ±20 °C.

In addition, Figure 8 indicates that the coating also helped preserve visual appearance and delay microbiological spoilage. While the control and the film without the active compound showed observable deterioration from day 5 onward, strawberries coated with active films, particularly Gel + *Ma*LAAO, showed no visible contamination throughout the analysis period, suggesting a surface antimicrobial effect acting synergistically with reduced water loss.

## 3. Discussion

Snake venoms have been shown to have antimicrobial potential, and different venoms have been evaluated against various groups of bacteria, especially those involved in infectious processes in humans. Snake venom proteins had demonstrated potent antibacterial properties that could serve as the basis for new therapeutic strategies [21]. There are some works that suggested activity against microorganisms of agriculture interest and as biotechnological tools to control. Alves demonstrated that ten crude venoms (seven from *Bothrop*s and three from *Crotalus*) and two purified toxins (gyroxin and crotamine) were promising approaches to control the phytopathogenic bacterium *Ralstonia solanacearum* [31]. Nunez et al. demonstrated antimicrobial activity of *B. asper* venom against *Xanthomonas* isolated from *Passiflora edulis* [32].

In this work, the results demonstrated antimicrobial activity of *Micrurus* venoms against various phytopathogenic bacterial species, showing growth inhibition higher against certain *Xanthomonas* species compared to *Pectobacterium* sp. or the fungus *Fusarium*. The compound responsible for the activity corresponds to LAAO in all venoms evaluated. In general, LAAOs in snake venoms catalyze the oxidative deamination of L-amino acids to α-keto acids, with the concomitant production of ammonia and hydrogen peroxide (H_2_O_2_). These enzymes have shown antimicrobial and cytotoxic activities. Furthermore, they are known to induce platelet aggregation and modulate immune system responses [33,34,35]. Despite their potential, LAAOs typically represent a small fraction of *Micrurus* venom, limiting their biotechnological exploration. In the Elapidae family, LAAOs typically account for approximately 5–8% of the total venom composition. However, in *Micrurus* species, their abundance is usually lower, rarely exceeding 7%. For instance, in *Micrurus mipartitus* and *M. dumerilli*, venom LAAOs account for approximately 4% and 3%, respectively [36,37]. The *M. ancoralis* proteome has yet to be described. However, according to the chromatography performed here, greater abundance was observed in the peak corresponding to LAAO than in the other venoms.

*X. axonopodis* and *X. perforans* showed major sensitivity to venoms. In the case of *X. campestris*, the lower sensitivity observed may be related to the robust oxidative stress defense systems described for this pathogen [38]. Similarly, *Pectobacterium* sp., as a Gram-negative bacillus with an outer membrane rich in lipopolysaccharides, could present an effective physical barrier that reduces the penetration and limits the direct action of protein toxins [39]. Conversely, the antifungal activity against *Fusarium* sp. required higher venom concentrations, indicating that this microorganism possesses robust defense mechanisms that mitigate the venom’s inhibitory effects. This increased resistance in filamentous fungi, compared to bacteria, is often attributed to the complexity of the fungal cell wall and efficient antioxidant systems [40]. Nevertheless, Salama et al. [41] reported significant inhibitory activity of L-amino acid oxidase (LAAO) purified from *Naja haje* venom against the phytopathogen *Fusarium proliferatum* at a concentration of 45 µg/well. These findings suggest that while *Fusarium* species exhibit a higher tolerance threshold, elapid-derived LAAOs remain promising candidates for controlling fungal growth when applied at appropriate doses.

Trapping snake venom in nanoparticles, more precisely in polymeric carriers such as chitosan or PLGA-based systems, represents a new strategy to enhance the therapeutic potential of venom components, especially for antibacterial applications. The approach has the benefit of enhancing stability and bioavailability [21]. In this work, the incorporation of crude *M. ancoralis* venom or their purified *Ma*LAAO into gelatin-based biofilms preserved the antimicrobial activity against *X. axonopodis* and *X. perforans*, with MIC values in the films very similar to those obtained for the enzyme in free solution. This result indicates that the polymeric matrix does not interfere with either the structure or the catalytic mechanism of LAAO. The use of biofilms as a delivery system for the antimicrobial components of *Micrurus* venom represents a strategy that offers multiple advantages for the preservation and controlled application of these bioactive compounds [42]. This biopolymeric matrix provides a stabilizing environment that protects the antimicrobial peptides and proteins of the venom against degradation caused by environmental factors such as temperature, pH, and light exposure, thereby maintaining their biological activity over extended periods [28].

All films exhibited a colorless characteristic, typical of gelatin-based films. The incorporation of the venom or fraction does not affect the clarity of the films (L*), significantly increases the yellow hue (b*), but does not significantly modify the overall color, maintaining visual characteristics suitable for applications in edible coatings and biodegradable films. According to Said and Sarbon [43], high L* values in gelatin films reflect a bright and uniform appearance, which coincides with this study’s findings. On the other hand, the increase in b* in the formulation with the venom and fraction suggests that the presence of bioactive proteins and peptides intensified the yellow hue, as also reported by Coyotl-Pérez et al. [44] in films enriched with natural additives.

The opacity of the films reflects the materials’ ability to reduce the transmittance of visible light, a relevant aspect in packaging applications that aim to protect food from radiation [27,45]. The opacity of the films increased markedly with the addition of *Ma*LAAO. These results are consistent with those reported by Coyotl-Pérez et al. [44] and Méndez et al. [30], who stated that the opacity of protein films depends not only on thickness but also on the internal organization of the matrix and the incorporation of bioactive compounds. Taken together, the data show that opacity is not determined solely by the concentration of gelatin but also by the microstructure of the material and the presence of external biomolecules, which can modify its optical properties and its functional potential as a packaging material.

The UV–visible light barrier correlates with opacity (Opa), a parameter that can indicate potential applications in foods, particularly those susceptible to lipid oxidation and light sensitivity. All films were practically transparent (Opa → 0), which is typical of gelatin-based films [27,30]. However, a slight increase in opacity and the UV–visible light barrier was observed in the Gel + *Ma*LAAO film, primarily due to the presence of the PBS buffer used to isolate LAAO from venom.

The FTIR spectra of the three analyzed formulations showed the characteristic absorption bands of gelatin-based biopolymeric materials. Direct comparison of the spectra did not reveal the appearance of new bands or significant shifts that could be unequivocally attributed to components of the venom or fraction. This suggests that, under the formulation and analysis conditions used, the incorporation of the venom or fraction did not produce detectable chemical interactions with the polymer matrix. This behavior is consistent with that reported in systems with complex biomolecules, where encapsulation within polymer networks restricts spectroscopic detection [30,46,47]. Taken together, these results support the hypothesis that the venom or its fraction is physically incorporated into the gelatin matrix without generating detectable structural modifications at the molecular level.

The thermal degradation pattern observed for the films is consistent with that typically reported for gelatin-based biopolymeric materials. The initial weight loss (76–121 °C) can be associated with the release of weakly bound water molecules, while the intermediate (140–280 °C) stage is mainly related to the degradation of glycerol and low-molecular-weight protein fractions present in the matrix. The final degradation (392–436 °C) stage is attributed to the breakdown of the main protein backbone of gelatin and associated peptide structures [48,49]. The relative loss of total mass was similar for the Gel and Gel + Ma formulations; however, a lower mass loss was evident for the Gel + *Ma*LAAO formulation, suggesting that the incorporation of bioactive proteins and peptides may have favored the formation of more stable structures against thermal degradation.

Scanning electron microscopy (SEM) shows no micropores or striations in the micrographs of the surface or cross-section of the Gel and Gel + Ma films, which means good compatibility between the venom and the gelatin matrix [28,30]. However, with the addition of *Ma*LAAO to the gelatin matrix, a possible segregation and phase separation occurs, possibly due to low compatibility of the gelatin with the phosphate-buffered saline (PBS—pH 7.2) in which the *Ma*LAAO fraction is solubilized. This is evident in the surface micrograph, where particles are clearly visible, and in the visual appearance of the film in the digital photographs. Since there are no striations or micropores in the cross-section of this film, it may be an indication that the fraction remained dispersed on the surface of the film and did not integrate into the polymer matrix. In similar studies, *Chlorella* and *Chlorella*-CNC films were prepared using phosphate basic saline (PBS) as a dispersing agent; the results showed that the surface of the films exhibited rough structures, including white circular features formed due to the agglomerated nanocrystals [50].

The results demonstrated the feasibility of developing antimicrobial biofilms incorporating LAAOs to extend the shelf life of strawberries, as evidenced by lower weight loss and improved visual appearance during storage at 20 °C compared with uncoated fruits and those coated with films lacking active compounds. Similar outcomes have been reported for gelatin-based edible coatings enriched with essential oils or other antimicrobial agents, which reduce decay incidence, maintain firmness, and stabilize physicochemical parameters in strawberries and other highly perishable fruits [42]. The biopolymeric films containing antimicrobial agents have shown activity against various phytopathogens responsible for the deterioration of fresh produce, while glycerol modifies film flexibility and barrier properties, thereby contributing to the maintenance of product quality during storage [51].

The reduced weight loss observed in treatments with active films can be attributed to a decrease in the respiration rate and moisture migration through the coated surface, effects previously described for gelatin-based biofilms and other polysaccharide coatings in both climacteric and non-climacteric fruits [52]. In addition, the progressive release of venom components—mainly LAAO—at the fruit–environment interface may generate microenvironments with local H_2_O_2_ concentrations sufficient to inhibit the growth of surface-spoilage fungi and bacteria, consistent with reports for other coating systems incorporating oxidizing agents or antimicrobial peptides [53,54]. These findings are particularly relevant given the highly perishable nature of strawberries, which is associated with their high moisture content, delicate structure, and susceptibility to microbial contamination, factors that lead to significant economic losses along the supply chain of this crop [3].

The present work provides new information on the inhibition of bacteria that affect crops, proposing LAAO as a substance with potential applications in biotechnological processes to improve phytopathogen control. Further studies should clarify the mechanisms involved in this process, and future applications should determine the maximum duration of protection and whether the biofilm is removed when the fruits or vegetables are washed.

## 4. Conclusions

Snake venom proteins exhibit diverse biological activities that could serve as the basis for new antimicrobial strategies in different areas. In this work, the antimicrobial activity of *Micrurus* venoms was demonstrated against various phytopathogenic microorganisms, such as *Xanthomonas* species and the fungus *Fusarium*. The isolated compound responsible for the observed antimicrobial activity was identified as L-amino acid oxidase (LAAO). Furthermore, a biofilm was prepared using this compound with the purpose of having a coating available that protects some perishable foods such as fruit, in this case strawberries, demonstrating that it is possible to obtain an antimicrobial film with LAAO that reduces the deterioration of this fruit, with the potential of possible use. In the same way, we provide new information on *Micrurus* venom and the control of plant pathogens.

## 5. Materials and Methods

### 5.1. Venoms

*Micrurus mipartitus*, *M. dumerilii*, and *M. ancoralis* venoms were obtained from adult specimens maintained in captivity at the institutional serpentarium of the University of Antioquia. All procedures were conducted under the Ministry of Environment genetic resource access permit RGE: 0156-15.

### 5.2. Antimicrobial Assay

#### 5.2.1. Bacteria

Antibacterial susceptibility assays were performed against the *Xanthomonas* genus obtained from *Passiflora edulis* fruit crops and isolated in previous work [32], following the methodology described by Vargas et al. [55], with some modifications. For the agar diffusion assay, the bacteria were cultured on Chocolate agar for 48 h. Subsequently, colonies were suspended in a sterile saline solution, which was adjusted to a 0.5 McFarland standard (absorbance at 600 nm), The bacterial suspension was then inoculated onto Mueller–Hinton agar plates (MERCK, Rahway, NJ, USA). In total, 10 μL of each *Micrurus* species venom diluted in saline solution was added to bacterial agar media and incubated for 24 h at room temperature (RT). A blank sterile saline solution instead of venom served as the negative control, and antibiotic–antimycotic solution (SIGMA-Aldrich, St. Louis, MO, USA) served as a positive control or reference. Diameters of the bacterial growth inhibition zones were measured using a ruler. Each assay was performed in duplicate.

The Minimum Inhibitory Concentration (MIC) was determined using the broth microdilution method. Serial dilutions of each venom (0.97 to 100 μg/mL) were prepared in a final volume of 50 μL of saline solution and transferred to 96-well plates; 100 μL/well of Mueller–Hinton broth was added, and then 50 μL of the bacterial inoculum was adjusted to a 0.5 McFarland standard (absorbance at 600 nm), added to each well, and incubated at RT for 24 h. Inhibition of bacterial growth was determined by measuring absorbance at 600 nm (Thermo Fisher Scientific, Waltham, MA, USA). The MIC was defined as the lowest venom concentration that completely inhibited visible microbial growth. Each dilution series included control wells, which consisted of 50 μL of saline solution or antibiotic (chloramphenicol 6 μg μL^−1^). All assays were performed in triplicate with internal duplicates.

Additionally, the venoms were also evaluated against *X. campestris* and *Pectobacterium* sp. following a similar protocol, with concentrations ranging from 9 to 150 μg/mL. The microplates were incubated at 37 °C for 4 h, and readings were performed by adding 20 µL of the dye 3-(4,5-dimethylthiazolyl-2)-2,5-diphenyltetrazolium bromide (MTT) at 0.8 mg/mL to each well, followed by incubation at 37 °C for 1 h to allow viable cells to metabolize the MTT. Finally, absorbance was measured at 660 nm using a Varioskan Lux multimode microplate reader (Thermo Fisher Scientific, USA), and the results were reported as the percentage of inhibition relative to the control [56].

#### 5.2.2. Fungus

Antifungal activity assays were performed against *Fusarium* sp. obtained from *Physalis peruviana* L. crops from the Eastern Antioquia subregion, Antioquia Department, Colombia [57].

The assays were conducted following the methodology described by Serrano et al. [58], with some modifications. A culture of *Fusarium* sp. previously grown on PDA (Potato Dextrose Broth agar) was used as a starting point. Each agar plate was flooded with Sabouraud broth, and the resulting suspensions were collected to prepare the inoculum. The conidia suspension was adjusted to an average concentration of 4 × 10^4^ spores, determined by counting in a Neubauer chamber. The assays were performed in 96-well plates using Sabouraud broth with 0.1% (*v*/*v*) Tween 20, previously sterilized, as the culture medium. The *M. mipartitus* and *M. dumerilii* venom were reconstituted in 40 µL of culture medium at different doses (200, 400, and 500 µg/mL) and were added to each well, followed by 110 µL of culture medium and 50 µL of the previously prepared inoculum, ensuring a final total volume of 200 µL per well. The tests were performed in duplicate for each treatment. Culture medium without inoculum (sterility) and others without venom (growing) were used as controls. The plate was incubated in constant darkness at 29 °C for 100 h. Absorbance measurements were performed in a microplate reader, with readings every 24 h at 490.

### 5.3. Isolating Bioactive Fractions from Micrurus Venom

First, 2 mg of lyophilized venom, dissolved in PBS (phosphate-buffered saline; 0.12 M NaCl, 40 mM sodium phosphate, pH 7.2) was separated on a Bio Sec-5 column (Agilent, Santa Clara, CA, USA, using a BioRad DuoFlow chromatograph (BioRad, Hercules, CA, USA), monitored at 215 nm. Elution was performed at 1 mL/min using the same buffer. Each fraction obtained was evaluated for antimicrobial activity in the agar diffusion assay, as previously described. To assess the purity of the active fraction, the proteins were submitted to analytical reverse-phase high-performance liquid chromatography (RP-HPLC, Shimadzu, Columbia, MD, USA) using a C_18_ column (Pinnacle, 5 mm particle diameter; 250 × 4.6 mm, RESTEK, Bellefonte, PA, USA), eluted at 1 mL/min with a linear gradient from 0 to 70% of solution B (0.1% TFA, 99.9% acetonitrile) in 25 min [59].

LAAO activity was determined using 5 µg of venom or bioactive fraction (in 10 µL of water) and added to 90 µL of a reaction mixture (250 mM L-Leucine, 2 mM o-phenylenediamine, and 0.8 U/mL horseradish peroxidase) in 50 mM Tris pH 8.0 buffer. After incubation at 37 °C for 60 min, the reaction was stopped with 50 µL of 2 M H_2_SO_4_, and absorbance was recorded at 492 nm [60]. The negative control consisted of the reaction buffer and substrate (L-leucine) in the absence of the enzyme.

### 5.4. Protein Identification by MS/MS Peptide Sequencing of Bioactive Fractions

The bioactive fraction was reduced with 5% 2-mercaptoethanol at 100 °C for 5 min and was run in a 12% SDS-PAGE gel [61]. The visible bands were excised from the gel and reduced with dithiothreitol and alkylated with iodoacetamide, followed by in-gel digestion with sequencing-grade trypsin in an automated processor (Intavis, Chicago, IL, USA) according to the manufacturer’s instructions. The resulting tryptic peptides were analyzed by nESI-MS/MS using a nano-Easy^®^ 1200 chromatograph in-line with a Q-Exactive Plus^®^ mass spectrometer (Thermo Fisher, Waltham, MA, USA).

Here, 5 μL of each tryptic digest was loaded on a C18 trap column (75 μm × 2 cm, 3 μm particle size), washed with 0.1% formic acid (solution A), and separated at a flow rate of 200 nL/min through a C18 Easy-spray^®^ column (15 cm × 75 μm, 3 μm particle size, Thermo Fisher, Waltham, MA, USA). Elution was carried out using a gradient towards solution B (80% acetonitrile, 0.1% formic acid) over 45 min (1–5% B in 1 min, 5–25% B in 30 min, 25–79% B in 6 min, 79–99% B in 2 min, and 99% B for 6 min). The mass spectra were acquired in positive mode at 1.9 kV, at a capillary temperature of 200 °C, using 1 μscan in the range 400–1600 m/z, maximum injection time of 100 ms, AGC target of 3 × 106, and a resolution of 70,000. The top 10 ions with 2–5 positive charges were fragmented with an AGC target of 1 × 105, a maximum injection time of 110 ms, a dynamic exclusion time of 5 s, and a resolution of 17,500. The resulting MS/MS spectra were processed against protein sequences contained in the UniProt/SwissProt Serpentes database (https://www.uniprot.org) using PEAKS X (Bioinformatics Solutions, Waterloo, ON, USA) and matches were assigned to known protein families by similarity. Cysteine carbamidomethylation was set as a fixed modification, while deamidation of asparagine or glutamine and methionine oxidation were set as variable modifications, allowing up to 3 missed cleavages by trypsin. Parameters for match acceptance were set to FDR < 0.1%, detection of at least one unique peptide, and −10lgP protein score ≥50. Tryptic peptides were de novo sequenced with assistance from PEAKS X (Bioinformatics Solutions).

### 5.5. Preparation and Characterization of Antimicrobial Films

Films were produced by drying film-forming solutions (FFSs) prepared with gelatin (1 g/100 g of FFS), glycerol (0.5 g/100 g of FFS), and either *M. ancoralis* crude venom (0.020 mg Ma/mL FFS) or its isolated bioactive fraction, *Ma*LAAO (0.016 mg *Ma*LAAO/mL FFS). The selection of *M. ancoralis* was based on its venom yield and high L-amino acid oxidase (*Ma*LAAO) content. To prepare the FFSs, gelatin was first hydrated in distilled water for 30 min at room temperature (RT) and subsequently solubilized at 55 °C for 30 min in a thermostatic bath. Once the gelatin was fully dissolved, glycerol and the respective venom component were added and homogenized via magnetic stirring [27]. Then, 10 g of the resulting FFS were dispersed in 5.5 cm diameter Petri dishes and allowed to dry at room temperature (±20 °C) until complete film formation. The thickness of the films was measured in at least 10 samples with a digital micrometer (±0.001 mm; Mitutoyo, Tokyo, Japan), taking the average of 10 different position measurements of each sample.

− *Antimicrobial Activity*

Films prepared with *M. ancoralis* venom or *Ma*LAAO were evaluated against the bacteria *Xanthomonas* and using agar and broth assays and the same conditions described previously to determine the MIC.

− *Moisture Content and Water Solubility*

Initially, film thickness was measured with a digital micrometer (supplied by Mitutoyo, Kawa-saki, Japan) at five distinct positions utilizing an accuracy of ±0.001 mm. The moisture content (MC) and water solubility (WS) values of the films were determined gravimetrically. In brief, the MC values were determined using a Precisa XM50 moisture balance (Precisa, Moosmattstrasse, Dietikon, Switzerland), and SM was determined by immersion in distilled water (50 mL) under mechanical agitation for 24 h at 25 ± C. After this period, the samples were dried (105 ± C, 24 h) and weighed. Both properties were calculated by weight difference with respect to the initial weight of the samples [27].

− *Color Parameters*

The color parameters of films—a* (red to green), b* (yellow to blue), L* (lightness), and ΔE* (total difference of color)—were determined using a colorimeter spectrophotometer CM-23d (supplied by Konica Minolta, Valencia, Spain) using the method SCI (Specular Component Included). The films were placed on a cell with a white background (L* = 31.73, a* = −1.11, and b* = 3.27), and the measurements were made directly on the surface. These analyses were carried out in triplicate from 5 random film measurements [30].

− *Opacity*

Opacity was determined using a UV–visible spectrophotometer (UviLine 9600 UV–vis spectrophotometer with SPECTRALAB 4.3 software supplied by SECOMAM SA, Alés, France). The thickness of each sample was measured at three points, and the films were cut (12 × 40 mm) and placed in the measuring cell. Readings were taken at 600 nm, and opacity was calculated using Opa = A/X, where A is the absorbance at 600 nm and X is the average film thickness (mm).

− *UV Light Transmission*

The ultraviolet/visible light barrier property was determined using a spectrophotometer (UviLine 9600 UV–vis spectrophotometer with SPECTRALAB software supplied by SECOMAM SA, Alés, Francia). Films were cut (12 × 40 mm^2^) and put in place of the cell to allow the passage of light. The measurements were made at wavelengths between 200 and 700 nm [30].

− *Fourier Transform Infrared (FTIR) Spectroscopy*

The films were cut into 12 × 40 mm^2^ rectangles. FTIR spectra were obtained in the range 4000–400 cm^−1^. The transmittance was recorded over 56 scans with a resolution of 4 cm^−1^ in a Jasco FT/IR-4X Spectrometer (instrument distributed by JASCO International Co., Ltd., Tokyo, Japan) [30].

− *Thermal Properties*

Thermogravimetric analysis was performed using a TGA PT 1000 instrument (LINSEIS, Selb, Germany). Samples were heated from 30 °C to 750 °C at a rate of 10 °C/min in an inert nitrogen atmosphere. These analyses allowed for the determination of thermal changes and comparison of the stability of the biopolymer films with respect to control samples.

− *Scanning Electron Microscopy (SEM)*

Film morphology was analyzed using the Dual-Beam Field Emission Scanning Electron Microscopy (FESEM) model Scios 2 LoVac (supplied by Thermo Fisher Scientific, USA) and the a scanning electron microscope model JEOL JSM 6490 LV (supplied by JEOL, Peabody, MA, USA), operating at an acceleration voltage of 0.8–10 kV and magnification of X1500–X15,000. The samples were covered with gold [30].

− *In Situ Film Tests*

To evaluate the potential of the film to protect food from antimicrobial contamination, strawberries were used, which were covered with the formulated film and subsequently evaluated for aspects such as weight loss and visual appearance.

− *Weight Loss*

Weight loss (WL) was determined by the gravimetric method reported by [27]. Strawberries were weighed at the beginning (W_i_) and end (W_f_) of the experiment. The relative weight loss (RWL) values were calculated as follows:

RWL = (W_i_ − W_f_)/W_i_ × 100

− *Visual Appearance*

To evaluate the film’s potential to protect food from microbial contamination, strawberries (groups of four) were covered with the formulated film and then evaluated for aspects such as weight loss and visual appearance (color, freshness, presence of spots, rot, and visible colonies of fungi or bacteria) by observation over a period of eight days (±20 °C, 55% RH). A group of strawberries covered with film without an active compound and another without film at all were used as the control, and those that did or did not present these aspects were counted in each group. The assays were performed two times.

## Figures and Tables

**Table 1:** Minimum Inhibitory Concentration (MIC) of *Micrurus mipartitus*, *M. dumerilii*, and *M. ancoralis* crude venoms against phytopathogenic bacteria. MIC values represent the lowest concentration of crude venom required to completely inhibit bacterial growth. Data are expressed as the mean ± SD of three independent experiments.

Venom	*X. axonopodis* (µg/mL)	*X. perforans* (µg/mL)
*M. ancoralis*	20.8 ± 7.2	18 ± 13
*M. dumerilii*	34 ± 14.2	28 ± 22
*M. mipartitus*	42 ± 12.6	75 ± 25

**Table 2:** Minimum Inhibitory Concentration (MIC) of isolated LAAOs from *Micrurus* venoms against *Xanthomonas*. Values represent the mean ± SD of three independent experiments (*n* = 9). Minimum Inhibitory Concentration (MIC) of LAAO isolated from *Micrurus mipartitus*, *M. dumerilii*, and *M. ancoralis* venoms against *Xanthomonas*. MIC values represent the lowest concentration of crude venom required to completely inhibit bacterial growth. Data are expressed as the mean ± SD of three independent experiments.

Fraction	*X. axonopodis* (µg/mL)	*X. perforans* (µg/mL)
*Ma*LAAO	13.5 ±1.8	16.7 ± 7.2
*Md*LAAO	7.2 ± 4.3	10.4 ± 10.8
*Mm*LAAO	18.7 ± 7.2	18.7 ± 10.8

**Table 3:** Physical properties of films: thickness, moisture content (MC), and water solubility (WS).

Films	Thickness (mm)	MC (%, Wet Basis)	SW (%)
Gel	0.046 ± 0.002 ^a^	11.7 ± 0.9 ^b^	30.1 ± 0.8 ^a^
Gel + *Ma*	0.046 ± 0.003 ^a^	10.8 ± 1.0 ^b^	31.8 ± 1.7 ^a^
Gel + *Ma*LAAO	0.051 ± 0.004 ^b^	8.3 ± 0.4 ^a^	31.4 ± 0.7 ^a^

^a,b^ Different letters within the same column indicate statistically significant differences between means according to Fisher’s Least Significant Difference (LSD) test (*p* < 0.05). Gel: film alone, Gel + Ma: film incorporated with *M. ancoralis* venom, and Gel + *Ma*LAAO: film incorporated with *M. ancoralis* LAAO.

**Table 4:** Opacity was determined using a UV–visible spectrophotometer. Color parameters (L*, a*, and b*) and opacity (Opa) of gelatin films added with Ma and *Ma*LAAO.

Films	L*	a*	b*	Opa
Gel	91.09 ± 1.68 ^a^	−0.20 ± 0.02 ^b^	4.92 ± 0.23 ^a^	1.30 ± 0.03 ^a^
Gel + Ma	90.56 ± 0.61 ^a^	−0.34 ± 0.12	5.15 ± 0.27	1.02 ± 0.13 ^a^
Gel + *Ma*LAAO	90.87 ± 0.39 ^a^	−0.46 ± 0.14 ^a^	5.68 ± 0.51 ^b^	7.30 ± 0.86 ^b^

^a,b^ Different letters in the same column indicate significant differences between the means obtained by Fischer’s test (*p* < 0.05).

## Data Availability

The original contributions presented in this study are included in the article/Supplementary Materials. Further inquiries can be directed to the corresponding authors.

## Supplementary Materials

The following supporting information can be downloaded at: https://www.mdpi.com/article/10.3390/toxins18060240/s1, File S1: Proteomic identification of isolated fractions via de novo MS/MS sequencing and multiple sequence alignment, confirming the presence of L-amino acid oxidases (LAAOs).
