# Supplementary material for: Antimicrobial Activity of Micrurus Venoms and Bioactive Films Functionalized with Purified L-Amino Acid Oxidase
Source: Toxins (Basel). 2026 May 22;18(6):240. doi: 10.3390/toxins18060240 (PMC13307728; doi:10.3390/toxins18060240)
Supplement: Supplementary file 1 [file toxins-18-00240-s001.zip › toxins-4260531-supplementary.pdf]

Supplementary Materials: Antimicrobial Activity of *Micrurus* Venoms and Bioactive Films Functionalized with Purified L-Amino Acid Oxidase

Vitelbina Núñez Rangel, Paola Rey-Suárez, Daniel Buitrago-Chinchilla, Laura Reyes-Méndez, Leidy Gómez-Sampedro, Alejandro Carmona-Jiménez, Mateo Rivillas-Ochoa and Adriana Muñoz-Bravo

Supplementary Material S1: Proteomic identification of isolated fractions via de novo MS/MS sequencing and multiple sequence alignment, confirming the presence of L-amino acid oxidases (LAAOs).

*Ma*LAAO

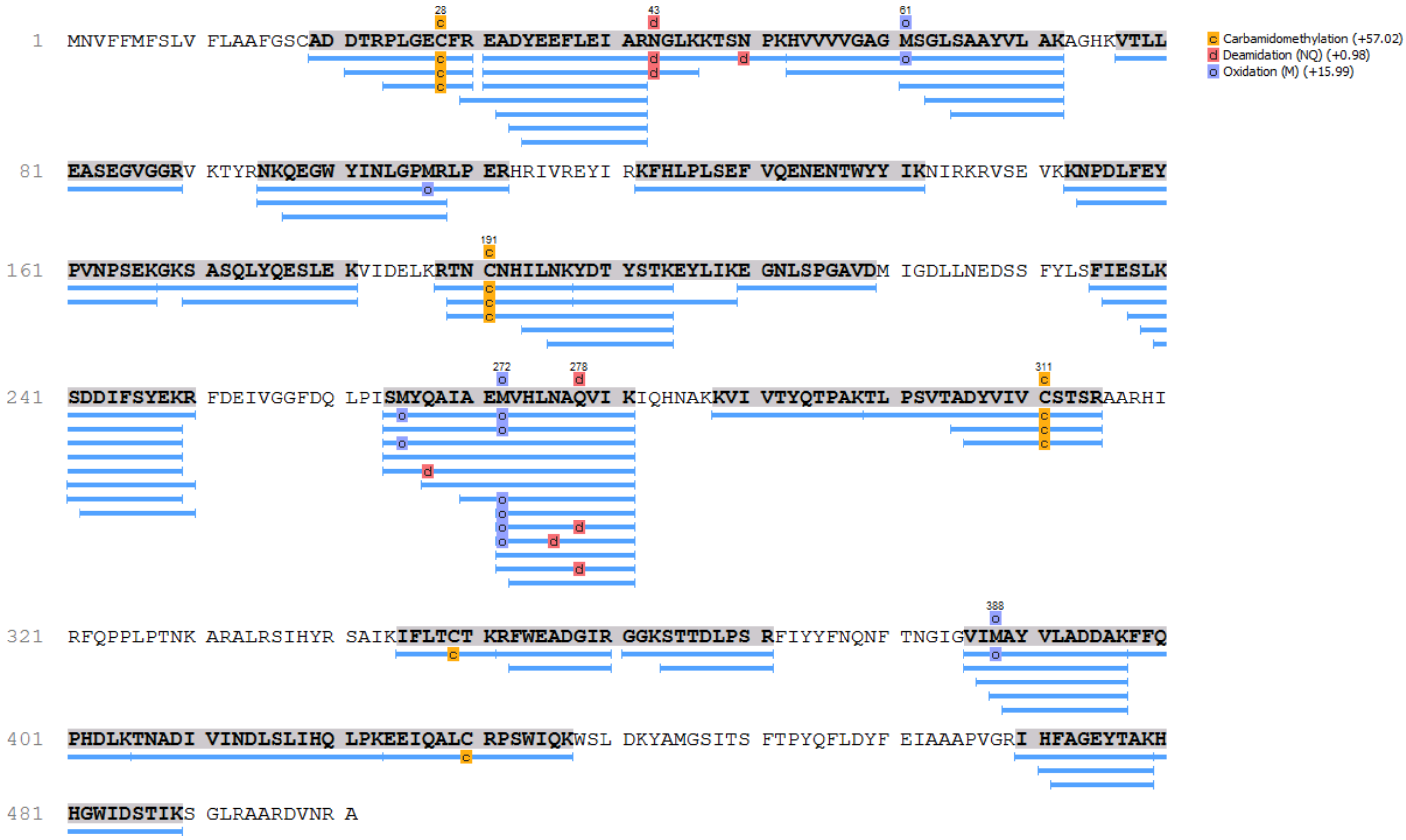

*Md*LAAO

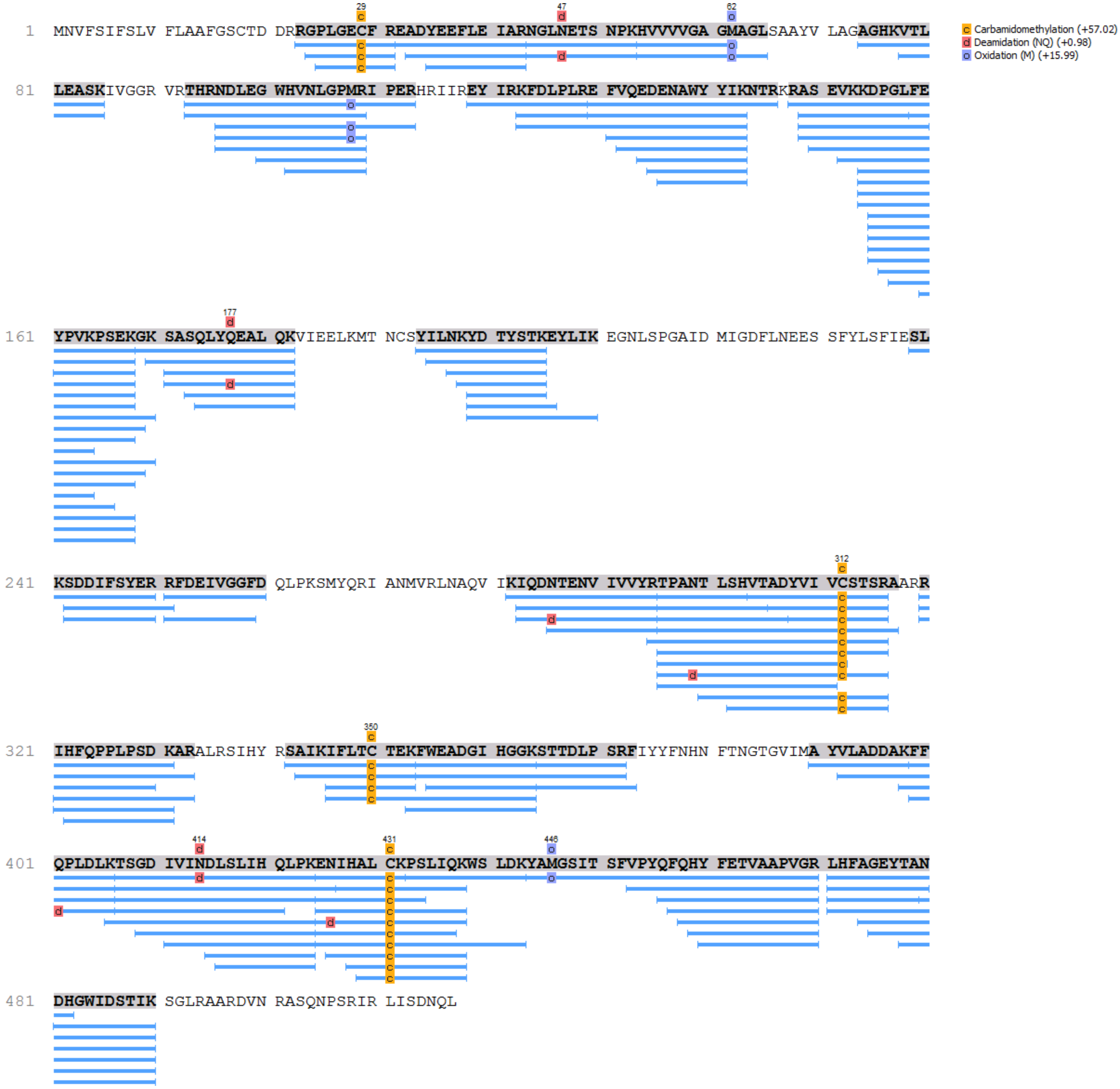

29 47 62
1 MNVFSIFSLV FLAAFGSCTD DRRGPLGECF READYEEFLE IARNGLNETS NPKHVVVVGA GMAGLSAAYV LAGAGHKVTL
81 LEASKIVGGR VRTHRNDLEG WHVNLGPMRI PERHRIIREY IRKFDLPLRE FVQEDENAWY YIKNTRKRAS EVKKDPGLFE
177
161 YPVKPSEKGK SASQLYQEAL QKVIEELKMT NCSYILNKYD TYSTKEYLIK EGNLSPGAID MIGDFLNEES SFYLSFIESL
312
241 KSDDIFSYER RFDEIVGGFD QLPKSMYQRI ANMVRLNAQV IKIQDNTENV IVVYRTPANT LSHVTADYVI VCSTSRAARR
350
321 IHFQPPLPSD KARALRSIHY RSAIKIFLTC TEKFWEADGI HGGKSTTDLP SRFIYYFNHN FTNGTGVIMA YVLADDAKFF
414 431 446
401 QPLDLKTSGD IVINDLSLIH QLPKENIHAL CKPSLIQKWS LDKYAMGSIT SFVPYQFQHY FETVAAPVGR LHFAGEYTAN
481 DHGWIDSTIK SGLRAARDVN RASQNPSRIR LISDNQL
c Carbamidomethylation (+57.02)
d Deamidation (NQ) (+0.98)
o Oxidation (M) (+15.99)

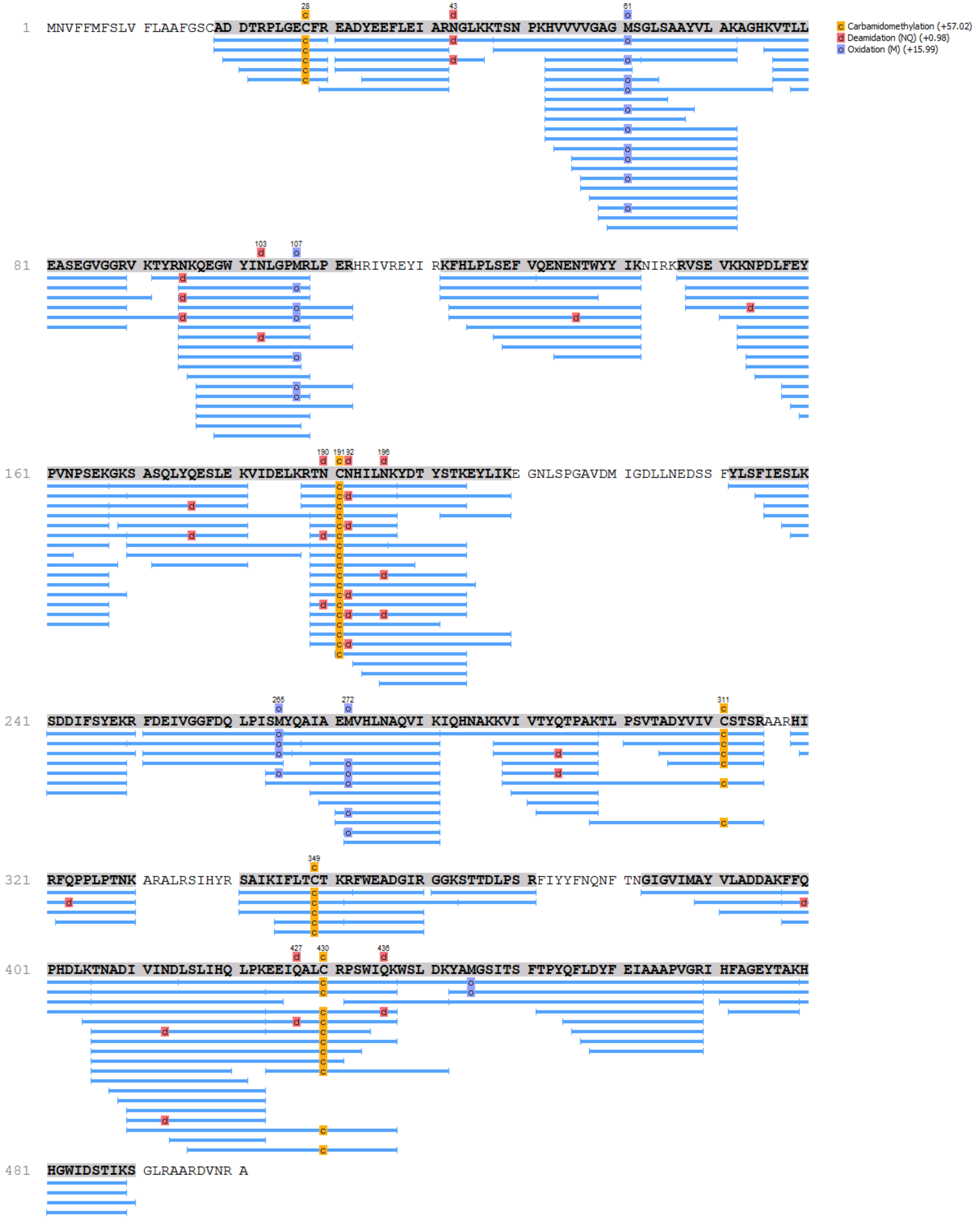
1 MNVFFMFSLV FLAAFGSCAD DTRPLGECFR EADYEEFLEI ARNGLKKTSN PKHVVVVGAG MSGLSAAYVL AKAGHKVTLL
81 EASEGVGGRV KTYRNKQEGW YINLGPMRLP ERHRIVREYI RKFHLPLSEF VQENENTWYY IKNIRKRVSE VKKNPDLFEY
161 PVNPSEKGKS ASQLYQESLE KVIDELKRTN CNHILNKYDT YSTKEYLIKE GNLSPGAVDM IGDLLNEDSS FYLSFIESLK
241 SDDIFSYEKR FDEIVGGFDQ LPISMYQAIA EMVHLNAQVI KIQHNAKKVI VTYQTPAKTL PSVTADYVIV CSTSRAARHI
321 RFQPPLPTNK ARALRSIHYR SAIKIFLTCT KRFWEADGIR GGKSTTDLPS RFIYYFNQNF TNGIGVIMAY VLADDAKFFQ
401 PHDLKTNADI VINDLSLIHQ LPKEEIQALC RPSWIQKWSL DKYAMGSITS FTPYQFLDYF EIAAAPVGRI HFAGEYTAKH
481 HGWIDSTIKS GLRAARDVNR A
c Carbamidomethylation (+57.02)
d Deamidation (NQ) (+0.98)
o Oxidation (M) (+15.99)

*Mm*LAAO
